# Psychometric properties of the intensive care delirium screening checklist when used by bedside nurses in clinical practice: a prospective descriptive study

**DOI:** 10.1186/s12912-020-00415-z

**Published:** 2020-04-10

**Authors:** Elke Detroyer, Annick Timmermans, Dana Segers, Geert Meyfroidt, Jasperina Dubois, Aimé Van Assche, Etienne Joosten, Koen Milisen

**Affiliations:** 1 Department of Public Health and Primary Care, Academic Centre for Nursing and Midwifery, KU Leuven, Kapucijnenvoer 35 – PB 7001/4, B-3000 Leuven, Belgium; 2 Department of Geriatrics, University Hospitals Leuven, Leuven, Belgium; 3 Department of Anesthesiology, Jessa Ziekenhuis, Hasselt, Belgium; 4 Department of Intensive Care, Ziekenhuis Oost Limburg, Genk, Belgium; 5 Department of Intensive Care Medicine, University Hospitals Leuven, Leuven, Belgium

**Keywords:** Delirium, Intensive care delirium screening checklist, Validation studies, Psychometrics, Sensitivity and specificity, User-friendliness

## Abstract

**Background:**

The Intensive Care Delirium Screening Checklist (ICDSC) demonstrates good psychometric characteristics in research settings. However, evidence about these characteristics in pragmatic ICU settings is inconsistent. This study evaluated psychometric properties and user-friendliness of the ICDSC when administered by ICU nurses in daily practice.

**Methods:**

This prospective study included 77 patients from a surgical intensive care unit. To examine the psychometric characteristics, the scores on the ICDSC (performed by bedside nurses) were compared with the scores on the Confusion Assessment Method for the Intensive Care Unit (CAM-ICU) (performed by researchers as gold standard). The user-friendliness was evaluated by 34 ICU nurses with a 20-item questionnaire.

**Results:**

The ICDSC had an area under the curve of 0.843. It showed a good diagnostic accuracy with a sensitivity of 81.0%, a specificity of 87.7%, and a 53.1% positive and 96.4% negative predictive value. The overall Cronbach’s alpha coefficient for all ICDSC scores was high (0.839). Overall, ICU nurses experienced the ICDSC as easy-to-use. The scale was usable in most surgical ICU patients. Yet, some nurses (11.8%) had problems to score the items ‘inappropriate speech’ and ‘symptom fluctuation’ in intubated patients.

**Conclusions:**

The ICDSC is a valid and user-friendly tool for delirium screening in daily ICU nursing practice. Yet, some problems were reported in intubated patients. Therefore, validation studies with specific focus on intubated patients are needed.

## Background

Delirium, an acute and/or fluctuating disturbance in attention and awareness, in combination with a change in perception or cognition, is a common and serious clinical syndrome in the intensive care unit (ICU) [1–3]. Delirium is associated with a poor functional recovery, an increased risk of dementia or mortality, a longer duration of mechanical ventilation, and a prolonged ICU or hospital length of stay [2–5]. Despite its importance, delirium is poorly noticed by healthcare professionals and its causes are thus undertreated [6–8]. Hence, routine delirium screening in ICU patients using a validated screening tool has been recommended [9].

Several screening tools for improving delirium recognition in the ICU have been developed. Based on systematic reviews [10–12] and the guidelines of the Society of Critical Care Medicine Pain, Agitation and Delirium (PAD) [9], the Confusion Assessment Method for the Intensive Care Unit (CAM-ICU) [13] and the Intensive Care Delirium Screening Checklist (ICDSC) [14] are advised for delirium screening in the ICU. Both tools are valid for delirium detection in ICU research settings. However, when CAM-ICU assessments were performed by nurses in clinical practice, the sensitivity of the tool is low which limits its use as a screening tool [15, 16]. Moreover, some additional disadvantages have been identified including the fact that CAM-ICU ratings are based on observations at one time-point using additional tests (i.e. attention screening examination) and the requirement for extensive training. The ICDSC with its good sensitivity (range, 64–99%, depending on the used cutoff values) and its continuous scoring system based on routine care observations, seems to be useful for delirium screening in daily practice [10, 12, 17]. Yet, lower sensitivity rates (range 43–47%) [15, 18] have been reported, and inconsistencies regarding its optimal cutoff [16, 17, 19, 20] and its use for screening and monitoring the severity of delirium in pragmatic ICU settings remain unclear [3, 8, 21, 22]. Furthermore, the user-friendliness of the ICDSC for bedside nurses in monitoring ICU patients – a prerequisite for implementation in practice - is unexplored. This study aimed to investigate the diagnostic accuracy, concurrent validity and internal consistency of the ICDSC when performed by ICU nurses in clinical practice. The user-friendliness of this scale in observing ICU patients during practice has been evaluated as secondary outcome.

## Material and methods

### Design, setting and sample

A prospective study was performed on an 18-bed surgical intensive care unit (ICU) of a general hospital in Belgium. Adult patients (18 years or older), Dutch speaking, and consecutively admitted to the hospital for an elective surgery with a planned ICU admission (enrolled during 6 months), were included. Those with severe sensory impairments (i.e. hearing or visual difficulties), neurosurgical indications and expected ICU discharge within 24 h were excluded. Written informed/proxy consent was obtained before study inclusion. Additionally, all ICU nurses of the participating unit were eligible for inclusion. The Medical Ethics Committee of the University Hospitals Leuven approved this study.

### Variables

#### Baseline measurements

Baseline data in patients, collected before surgery using patient interview or based on chart review, were age, sex, marital status, education level, social living circumstances, type of surgery, number of medications, cognitive functioning and presence of confirmed dementia. Cognitive functioning was measured using the 12-item Mini-Mental State Examination (MMSE) [23]. The total sum score varies between 0 and 12, in which lower scores indicate poorer cognitive functioning.

Nurses’ data including age, sex, education level, work experience as a nurse and delirium training received the last 5 years, were collected through a questionnaire.

#### Delirium and delirium severity

Delirium was both measured with the Intensive Care Delirium Screening Checklist (ICDSC) [14] and the Confusion Assessment Method for the intensive Care Unit (CAM-ICU) [13]. The ICDSC contains 8 items, including level of consciousness; inattention; disorientation; hallucinations; psychomotor activity; speech or mood disturbance; sleep disturbance; and fluctuation of symptoms, which were scored based on observations during each 8-h shift [14]. The level of consciousness was scored as (a) no response/coma, (b) vigorous stimulation/stupor, (c) drowsiness, (d) wakefulness, or (e) hypervigilance. In comatose or stuporose patients, there was no further delirium evaluation during that period. Only patients who were awake were considered as having a normal consciousness, and received no points on that item. The other seven items were rated as absent (0) or present (1), resulting in a total score ranging between 0 and 8. A score of 4 or more indicates delirium [14]. The ICDSC was translated into Flemish by three of the authors (ED, AT, DS), and examined by another member of the research team (KM) and two Dutch-speaking external clinical experts with medical and psychological backgrounds. They all had good knowledge of English and an extensive expertise in delirium.

The CAM-ICU is a diagnostic algorithm for delirium, which was completed based on a cognitive assessment using questions with nonverbal answers and simple commands (e.g. Attention Screening Examination). Delirium was diagnosed when there was a positive rating on the criteria acute onset OR fluctuation, AND inattention AND altered level of consciousness OR disorganized thinking [13]. The level of consciousness was evaluated using the Richmond Agitation-Sedation Scale (RASS) [24]. This scale ranges from − 5 (unarousable) to + 4 (combative). In patients with RASS-score − 5 or − 4, there was no further delirium evaluation at that moment.

The severity of delirium was evaluated using the short form of the Confusion Assessment Method Score for delirium severity (CAM-S) [25], including the four core criteria for delirium. The items ‘inattention’, ‘disorganized thinking’ and ‘altered level of consciousness’ were scored as absent (0), mild (1) or marked (2), the item ‘acute onset or fluctuating course’ as absent (0) or present (1) [25]. The total sum score varies between 0 and 7, in which higher scores indicate a greater severity.

#### User-friendliness of the ICDSC

The user-friendliness of the ICDSC for the bedside nurses was measured with a 20-item questionnaire (see Table 4 for all aspects measured), and of which the details are reported elsewhere [26, 27].

### Procedure

Patients were recruited by one of the three study nurses on the evening before surgery. Afterwards, patient baseline data were collected. Delirium was measured during the first 10 days of ICU hospitalization by bedside and study nurses. Those measurements were performed independently, blinded to the ratings of each other. Bedside nurses administered the ICDSC to score delirium on a twice daily basis (i.e. morning and evening shift). Study nurses performed four assessments (i.e. on postoperative days 2, 3, 5, 9) in enrolled patients, unless patients had an earlier ICU discharge. The assessments took place during the same 8-h shift of the bedside nurses’ ratings, including the performance of the CAM-ICU and CAM-S, as described above. Those CAM-ICU and CAM-S assessments were considered as gold standard. At the end of the patient enrollment period, the bedside nurses received a questionnaire to collect their baseline characteristics and the user-friendliness of the ICDSC. The completion and return of those questionnaire was seen as informed consent.

Both bedside and study nurses were educated in administering the instruments (i.e. CAM-ICU or ICDSC) by two experts in delirium (ED and KM). Study nurses were trained using the manual of CAM-ICU. The education also included the assessment of clinical cases in real practice and follow-up discussion. Interrater reliability for the CAM-ICU (i.e. evaluated in a random sample of 12 paired observations in patients) was κ = 1.00, which indicates perfect agreement. Bedside nurses were trained to rate the ICDSC during a baseline 1-h course followed by three follow-up sessions.

### Statistical analysis

To explore patient and nursing data including the data about the user-friendliness of the ICDSC, descriptive analysis (i.e. mean or median and standard deviation or interquartile ranges for continuous data, absolute number and percentages for categorical data) were used.

Paired delirium ratings of bedside and study nurses were evaluated to examine: 1) the diagnostic accuracy of the ICDSC for the CAM-ICU, 2) the level of agreement between the ICDSC and the CAM-ICU, and 3) the concurrent validity between the ICDSC and the CAM-S. Diagnostic accuracy of the ICDSC was explored based on the receiver operating characteristic (ROC) curve in combination with the sensitivity, specificity, positive and negative predictive values for the different cutoffs of the ICDSC. Additionally, “delirious” (positive CAM-ICU and ICDSC score ≥ 4) and “non-delirious” (negative CAM-ICU and ICDSC < 4) patient groups were evaluated using the Cohen’s kappa coefficients (κ), the proportion of observed agreement (P_0_), prevalence index (PI) and bias index (BI). The P_0_ express the ratio of exact agreement between the two assessment methods (i.e. CAM-ICU and ICDSC) per total number of assessments, while the κ corrects for chance [28]. Yet, as explained in literature, contradictions in P_0_ and κ can be seen due to prevalence and bias effects [28]. The concurrent validity between the ICDSC and CAM-S scores was examined with the Spearman’s rho correlation coefficient (i.e. for the total group, and for the delirious and non-delirious group separately).

Finally, internal consistency of the ICDSC was explored with the Cronbach’s alpha and item-total correlations on all ICDSC scores together.

All analysis were two-sided and performed using SPSS version 17.0 (SPSS Inc., Chicago, IL). *P*-values < 0.05 were considered as significant.

## Results

### Study sample

**Fig. 1:**
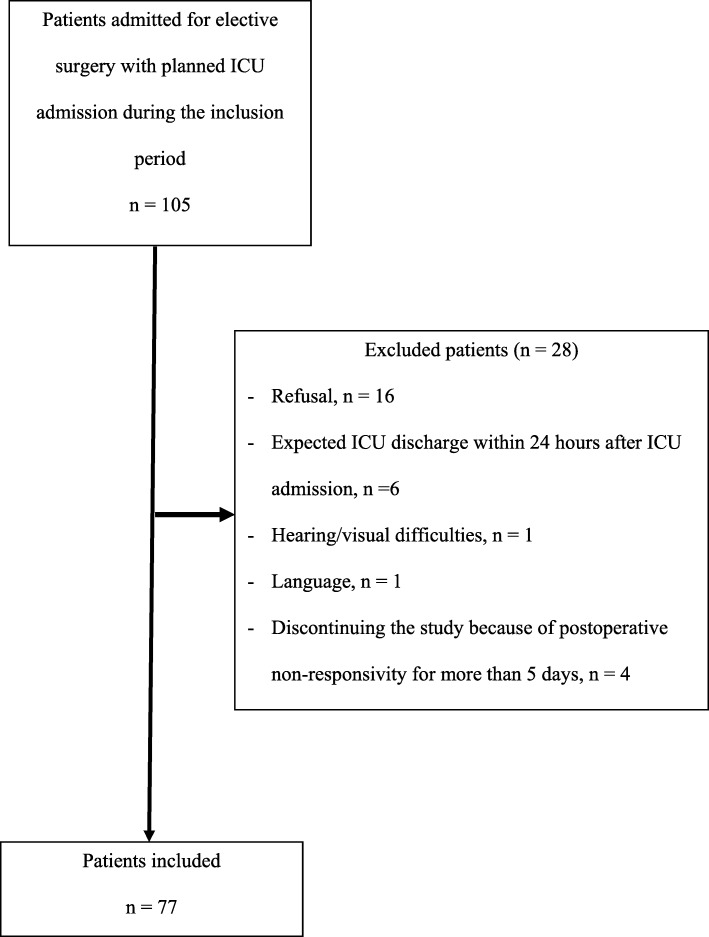
Overview of the study sample in patients

One hundred and five patients were consecutively admitted to the hospital for elective surgery with a planned ICU admission. Sixteen of them refused to participate, and another 12 were excluded because there was an expected ICU discharge within 24 h after ICU admission (*n* = 6), because of severe hearing or visual difficulties (*n* = 1), or inability to understand Dutch (n = 1). Four patients discontinued the study because they were postoperative not responsive for more than 5 consecutive days (Fig. 1). A total of 77 patients were included in which their baseline data are shown in Table 1. The majority of patients were admitted for coronary artery bypass grafting (CABG) (*n* = 44, 57.1%) (Table 1).

**Table 1:** Baseline data of included patients (*n* = 77)

Characteristics	
Age, median years (IQR)	72 (13)
Sex, n (%)	
Female	14 (18.2)
Male	63 (81.8)
Marital status, n (%)	
Married	59 (76.6)
Single	2 (2.6)
Widowed	13 (16.9)
Divorced	3 (3.9)
Education level, n (%)	
Low (< 15 years)	37 (48.0)
Moderate (15–18 years)	23 (29.9)
High (> 18 years)	17 (22.1)
Social living circumstances before admittance to the intensive care unit, n (%)	
At home	75 (97.4)
Service flat	1 (1.3)
Residential facilities	1 (1.3)
Type of surgery, n (%)	
CABG	44 (57.1)
Valve replacement	15 (19.5)
Combination valve replacement and CABG	12 (15.6)
Thorax surgery	3 (3.9)
AAA	3 (3.9)
Number of medications, median (IQR)	4 (2.0)
Confirmed diagnosis of dementia, n (%)	1 (1.3)

A maximum of 1540 ICDSC (=77x2x10) and 308 CAM-ICU (=77 × 4) observations were expected to be performed. However, because of a shorter ICU stay or unresponsiveness of included patients during study participation, 508 ICDSC and 168 CAM-ICU observations were completed, generating 143 paired observations. For 25 paired observations, delirium assessments were not performed during the same 8-h shift; and therefore excluded from further analyses.

Of the 49 bedside nurses, 34 of them filled out the questionnaire (i.e. response rate = 69.4%). Their mean age was 29.8 years (±SD 6.3 years). Most of the nurses were female (*n* = 27, 79.4%), had bachelor degree alone (*n* = 11, 32.4%) or with an additional degree in intensive care (*n* = 17, 50.0%), and had more than 6 years of work experience on the intensive care unit (*n* = 16, 47.1%). Only 4 nurses (12%) followed a training in delirium for the last 5 years.

### Post-operative delirium

Postoperative delirium (i.e. minimum one positive CAM-ICU score) occurred in 17 of the 77 patients (22.1%), or in 21 of the 143 paired observations (14.7%). According to the ICDCS, 104 of the 508 observations (20.5%) presented an overall ICDSC score of 4 or more, representing a possible delirium.

### Diagnostic accuracy (Table 2)

**Fig. 2:**
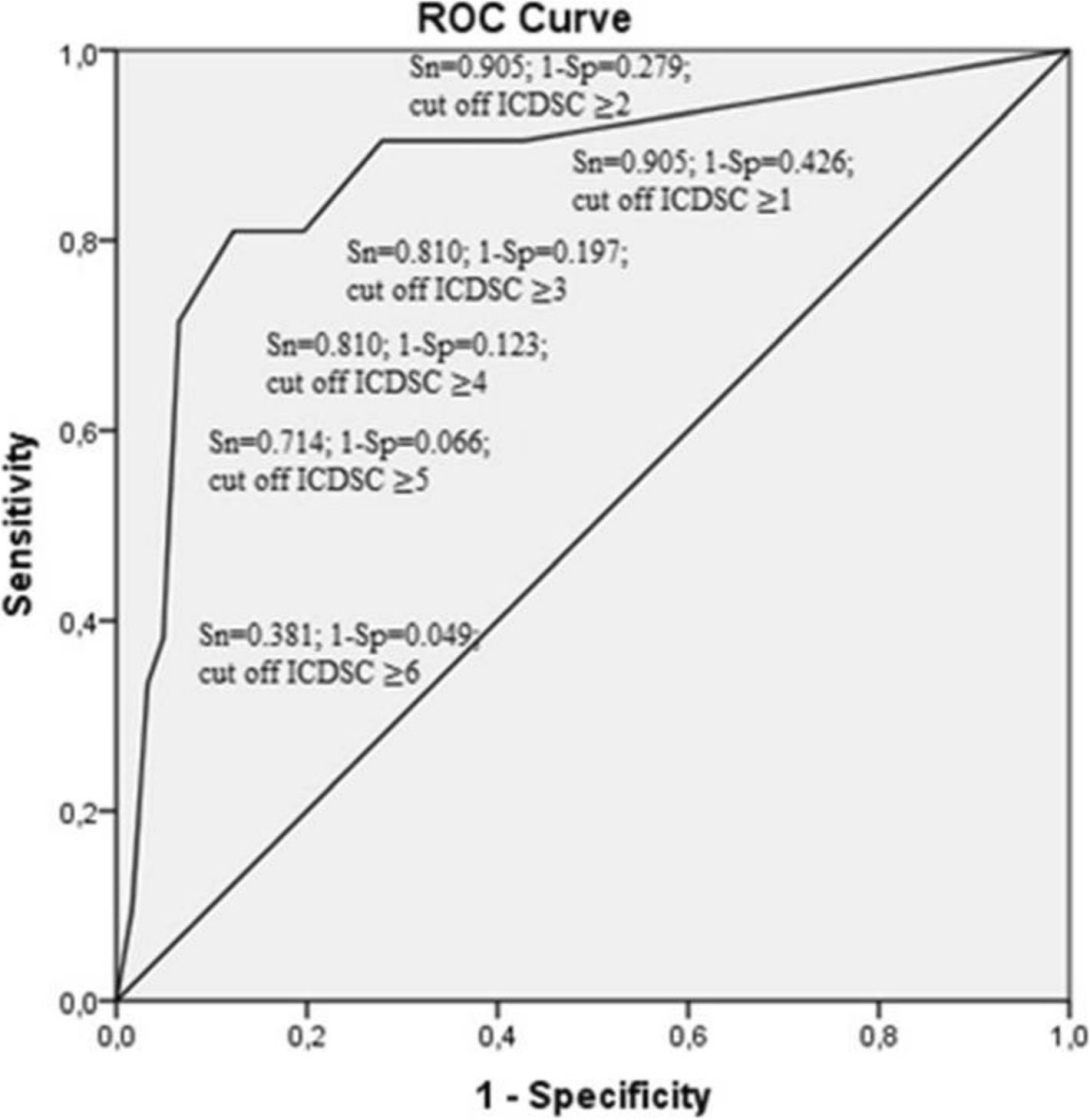
ROC curve of the ICDSC scores compared with the CAM-ICU as reference standard. ROC: receiver operating characteristic; ICDSC: Intensive Care Delirium Screening Checklist; CAM-ICU: Confusion Assessment Method for the Intensive Care Unit; Sn: sensitivity; Sp: specificity

The ICDSC yielded an area under the ROC curve of 0.873 (95% confidence interval (CI): 0.779–0.966) (Fig. 2). The diagnostic accuracy of the ICDSC with the original cutoff of 4 was good, with 81.0% sensitivity and 87.7% specificity. Bedside nurses detected 17 true-positive delirium observations, 4 false-negative and 15 false-positive observations. This results in a median positive predictive value and a high negative predictive value (Table 2). Lowering the cutoff point to 3 did not increase the sensitivity but reduced the specificity. Increasing the cutoff point to 5 reduced the sensitivity and increased the specificity (Table 2).

**Table 2:** Diagnostic accuracy of the ICDSC administered by bedside nurses for the CAM-ICU (study nurses) as gold standard in 143 paired observations

Instruments	Cutoff	Sensitivity % (95% CI)	Specificity % (95% CI)	PPV % (95%CI)	NPV % (95% CI)	Accuracy % (95% CI)
Positive CAM-ICU						
ICDSC	2	90.5 (71–97)	72.1 (64–79)	35.8 (24–49)	97.8 (92–99)	74.8 (67–81)
	3	81.0 (60–92)	80.3 (72–86)	41.4 (28–57)	96.1 (90–98)	80.4 (73–86)
	4	81.0 (60–92)	87.7 (81–92)	53.1 (36–69)	96.4 (91–99)	86.7 (80–91)
	5	71.4 (50–86)	93.4 (88–97)	65.2 (45–81)	95 (90–98)	90.2 (84–94)

### ICDSC and CAM-ICU agreement

Agreement in defining delirious and non-delirious patients was moderate (P_0_ = 0.87; κ = 0.56, 95% CI: 0.38–0.74, *p* < 0.001), with prevalence and bias indexes of 0.63 and 0.08, respectively.

### Concurrent validity ICDSC with CAM-S

Correlation between paired ICDSC scores with CAM-S scores was moderate both for the total group (r_spearman_ = 0.68, p < 0.001) as for the non-delirious subgroup (r_spearman_ = 0.54, *P* < 0.001). A non-significant correlation between both scales (r_spearman_ = 0.41, *p* = 0.06) was seen within the delirious group (21 paired observations).

### Internal consistency

The overall Cronbach’s alpha coefficient for all the ICDSC scores was 0.839. The alpha coefficients if one of the items was deleted ranged between 0.808 and 0.837 (Table 3). The items correlated strongly (i.e. items 1, 5, 8) (r_Pearson_ = 0.604–0.661) to moderately (i.e. items 2, 3, 4, 6, 7) (r_Pearson_ = 0.469–0.588) with the sum of the other items (Table 3).

**Table 3:** Pearson item-total correlation coefficients of the ICDSC (*n* = 77 patients; 507 test occasions)

ICDSC items	Corrected item-total correlations	Total alpha if item is deleted
Item 1 “Altered level of consciousness”	0.638	0.811
Item 2 “Inattention”	0.586	0.819
Item 3 “Disorientation”	0.588	0.819
Item 4 “Hallucination, delusion, psychosis”	0.507	0.829
Item 5 “Psychomotor agitation or retardation”	0.661	0.808
Item 6 “Inappropriate speech or mood”	0.575	0.822
Item 7 “Sleep/wake cycle disturbance”	0.469	0.837
Item 8 “Symptom fluctuation”	0.604	0.816

### User-friendliness

Most respondents mainly or entirely agreed that the concepts of the ICDSC items are clear (*n* = 33, 97.1%) and compatible with the language used in practice (*n* = 32, 94.2%). Furthermore, most of them agreed that the way in which the items are defined is free of values and judgement (*n* = 28, 82.4%), and differences in the response options of the items are mainly or entirely clear (*n* = 29, 85.3%). Although most nurses mainly or entirely agreed that the ICDSC items in themselves are clear, one nurse mainly disagreed for items 3, 4, 5 and 7 (Table 4), and four nurses mainly disagreed for items 6 (inappropriate speech or mood) and 8 (symptom fluctuation). Rating the two latter items gave problems in intubated patients. Although all nurses mainly or entirely agreed that they had sufficient knowledge from training and experience to evaluate the items on the ICDSC, eleven (32.4%) of them indicated they required help from others to rate the scale. Furthermore, some nurses disagreed that a quickly choice between the possible answers (*n* = 4, 11.8%) could be made or that the instructions facilitated to choose the correct answers (*n* = 1, 2.9%). Although 28 nurses (82.4%) mainly or entirely agreed that the ICDSC is a handy instrument to use in practice, 14 nurses (41.2%) mainly disagreed that the instrument adds value to their daily practice. Last, 7 nurses (20.6%) completed the ICDSC ratings in less than 1 min, 23 nurses (67.6%) in 1 to 2 min and 4 nurses (11.8%) in 3 to 5 min.

**Table 4:** User-friendliness of the ICDSC (*n* = 34 bedside nurses of the ICU)

Items	Entirely disagree, n (%)	Mainly disagree, n (%)	Mainly agree, n (%)	Entirely agree, n (%)
Clarity of content/concepts of the scale				
The concepts of the scale were clear to me	0 (0)	1 (2.9)	16 (47.1)	17 (50.0)
The concepts were compatible with the language used in practice	1 (2.9)	1 (2.9)	13 (38.2)	19 (55.9)
The way in which the observations are described is free of values and judgement	0 (0)	6 (17.6)	11 (32.4)	17 (50.0)
There was a clear difference between the possible answers	0 (0)	5 (14.7)	15 (44.1)	14 (41.2)
Nurses’ perception of their competence to fill out the scale				
I have sufficient knowledge from my training/experience to evaluate the observations on the scale	0 (0)	0 (0)	14 (41.2)	20 (58.8)
I could quickly make a choice between the possible answers	0 (0)	4 (11.8)	17 (50.0)	13 (38.2)
I requested help from others because it was not clear to me what was being asked	11 (32.4)	12 (35.3)	7 (20.6)	4 (11.8)
The instructions on the form helped me in choosing the answers	0 (0)	1 (2.9)	15 (44.1)	18 (52.9)
Relevance/feasibility of the scale				
I found it a handy instrument to spot delirium symptoms	0 (0)	6 (17.6)	21 (61.8)	7 (20.6)
This instrument offered added value to my practice of nursing	0 (0)	14 (41.2)	12 (35.3)	8 (23.5)
Clarity of single ICDSC items				
Item 1 (altered level of consciousness) is clear to me	0 (0)	0 (0)	13 (38.2)	21 (61.8)
Item 2 (inattention) is clear to me	0 (0)	0 (0)	12 (25.8)	22 (64.7)
Item 3 (disorientation) is clear to me	0 (0)	1 (2.9)	10 (29.4)	23 (67.6)
Item 4 (hallucination, delusion, psychosis) is clear to me	0 (0)	1 (2.9)	15 (44.1)	18 (52.9)
Item 5 (psychomotor agitation or retardation) is clear to me	0 (0)	1 (2.9)	13 (38.2)	20 (58.8)
Item 6 (inappropriate speech or mood) is clear to me	0 (0)	4 (11.8)	14 (41.2)	16 (47.1)
Item 7 (sleep/wake cycle disturbance) is clear to me	0 (0)	1 (2.9)	14 (41.2)	19 (55.9)
Item 8 (symptom fluctuation) is clear to me	0 (0)	4 (11.8)	13 (38.2)	17 (50.0)

## Discussion

Although the ICDSC has been advised for delirium screening in the ICU, evidence about its test characteristics with different cutoffs and user-friendliness when performed by bedside nurses is inconsistent [3, 8, 14, 16, 17, 19–22] This study presents evidence to support the psychometric properties and user-friendliness of the ICDSC used by nurses in clinical practice.

The ICDSC is able to make a good differentiation between patients with and without delirium as compared to the CAM-ICU as reference method. It had good sensitivity and specificity rates when the original cutoff point was used. In contrast with two previous studies [19, 20], lowering the cutoff to 3 would not affect the detection of delirious patients, yet would increase the number of false positives. Increasing the cutoff to 5 would detect less delirious patients, however decrease the number of false positives. Since the ICDSC is used for delirium screening, the original cutoff of 4 remains a good threshold for use in daily ICU practice. The sensitivity is consistent with the validation studies conducted in the research settings (75–99.0%) [16, 17, 19], where a limited number of trained researchers administered the ICDSC. However, compared to the studies evaluated in daily practice (43–71.9%) [8, 18, 20–22], sensitivity in our study was higher. This discrepancy may be due to the lack of training [21], the use of a different reference standard [8, 19, 21–23] or caused by the inclusion of other types of ICU patients (e.g. neurosurgery and/or medical (oncology) patients) [8, 18, 21] in these studies.

Agreement between the CAM-ICU and ICDSC was further evaluated with kappa statistics, showing a moderate kappa despite the high observer agreement between both instruments. This difference reflects bias by homogeneity of the sample (prevalence index = 0.63) which reduces the kappa coefficient. However, importantly, the magnitude of kappa was not influenced by a systematically different classification method between the two scales (bias index = 0.08).

The concurrent validity of delirium severity between the ICDSC and the CAM-S was moderate, yet, correlations within the subgroups of nondelirious and delirious patients separately were somewhat lower. Yet, for use as severity instrument in delirious patients, further research testing that specific aspect is necessary. Indeed, the ICDSC was only tested against the CAM-S, which may be insufficiently extensive to evaluate delirium severity. On the other hand, the long form of the CAM-S includes - against the four core items - also the items disorientation, memory impairment, perceptual disturbances, psychomotor agitation/retardation and altered sleep-wake cycles, items also found in the ICDSC [25]. Therefore, further research testing the ICDSC against the full version of the CAM-S is necessary.

Furthermore, reliability analysis revealed good internal consistency with a value which is in line with those in previous studies (0.72–0.86) [19, 21]. Overall, all items showed good item-total correlations and seemed to be worthy of retention.

In general, the user-friendliness of the ICDSC was evaluated as good. Yet, important findings about the ICDSC were identified. First, regarding the clarity of the individual items, none of those items were evaluated as entirely clear for all nurses but, unfortunately, no comments about the perceived difficulties were given. However, four nurses commented that the items ‘inappropriate speech or mood’ and ‘symptom fluctuations’ were difficult to rate in intubated patients. Therefore, these nurses rated the two items as mainly unclear. Hence, we can assume that the difficulties with these two items were not associated with the concepts but with their use in a subpopulation of non-verbally active ICU patients. Yet, one could argue that using the ICDSC in intubated patients affects its psychometric properties. Indeed, a previous study [8] revealed that its sensitivity was lower in a subgroup of non-verbally active patients compared to those in the verbally active subgroup. However, because of the low amount of intubated patient observations in our study (*n* = 12), sensitivity analysis in this subgroup was not performed. Hence, more research on the ICDSC’s psychometric properties within different subgroups of ICU patients is needed. Second, although all nurses indicated to have appropriate knowledge from training and clinical experience to score the ICDSC items, almost one third indicated the need for help to rate the scale. The reason for this discrepancy cannot be determined as no information regarding the content of the requested help was available. Yet, it indicates that the implementation of the ICDSC in daily practice require more than a simple educational session. Indeed, a comprehensive training session, not only before but also educational support during the implementation process is necessary.

Last, a small majority of nurses agreed that the ICDSC adds value to their nursing practices. One possible reason could be that screening without further action is useless. Indeed, screening should be part of a global delirium management protocol which was not implemented in this study. Because of the small sample size, we were not able to compare the characteristics of nurses who agreed versus those who disagreed. Nevertheless, the importance of delirium evaluation with a screening instrument is well established. Delirium screening based on clinical impressions showed inferior sensitivity compared to screening with a screening tool [8]. Hence, this highlights the need for nursing education about the importance of standard delirium screening with screening tools and its implementation in daily practice. The optimum types of educational strategies should be explored in further research.

Some methodological limitations need to be indicated. First, one might criticize the reference standard we used to diagnose delirium; e.g. the CAM-ICU algorithm rated by the study nurses and not the criteria of the Diagnostic and Statistical Manual of Mental Disorders (5th ed.; DSM-V) evaluated by an experienced physician. Yet, the reliability and validity of this reference standard was warranted because all research nurses followed an extensive training session in delirium using a validated diagnostic model also used in prior research [27, 29, 30], and has been shown to achieve high quality delirium assessments [31]. Second, the analysis regarding accuracy, agreement and validity were based on paired observations in 77 patients, which were not independent. Despite the fact that this might have potentially affected the results, the study was descriptive and not inferential. Yet, it is not expected to be extensively influenced by non-independence. Third, the paired delirium ratings of bedside and research nurses were not performed at the same point in time, which might result in bias because of the fluctuating nature of delirium. Yet, since there are differences in the methods of scoring within the used instruments; ICDSC ratings are scored with observations from the previous 8 h and CAM-ICU/CAM-S ratings are scored with observations made at one time point; evaluating delirium with both methods at the same time was impossible. Yet, by including only the assessments which were made within the same 8-h shift in those analysis, we were able to reduce the time span between the two methods used. Last, one might criticize the used technique for the ICDSC translation into Flemish. However, no gold standard exists [32–34]. Instead of performing a back-translation, an expert panel with expertise in delirium was used to check the quality of the translation. This technique was successfully used in previous studies [32–34], and is considered to be more effective for ensuring that the translation is performed appropriately [33, 34].

## Conclusions

The ICDSC seems to be a valid and user-friendly tool for delirium screening in clinical ICU nursing practice. However, further validation studies with specific focus on intubated patients and on the aspect of monitoring delirium severity are required.

## Data Availability

The datasets used and analyzed during the current study are available from the corresponding author on reasonable request.

## Abbreviations

ICU: Intensive care unit
CAM-ICU: Confusion Assessment Method for the Intensive Care Unit
ICDSC: Intensive Care Delirium Screening Checklist
MMSE: Mini-Mental State Examination
RASS: Richmond Agitation-Sedation Scale
CAM-S: Confusion Assessment Method Scale for delirium severity
ROC: receiving operating chacteristic curve
P0: Proportion observed agreement
PI: Prevalence Index
BI: Bias Index
CABG: Coronary artery bypass grafting
SD: Standard deviation
CI: Confidence interval
AUC: Area under the curve
DSM-V: Diagnosis and Statistical Manual of Mental Disorders 5th edition
